# Genotyping of *Plasmodium vivax* Reveals Both Short and Long Latency Relapse Patterns in Kolkata

**DOI:** 10.1371/journal.pone.0039645

**Published:** 2012-07-13

**Authors:** Jung-Ryong Kim, Amitabha Nandy, Ardhendu Kumar Maji, Manjulika Addy, Arjen M. Dondorp, Nicholas P. J. Day, Sasithon Pukrittayakamee, Nicholas J. White, Mallika Imwong

**Affiliations:** 1 Faculty of Tropical Medicine, Department of Clinical Tropical Medicine, Mahidol University, Bangkok, Thailand; 2 Centre for Tropical Medicine & Parasitology, Calcutta, India; 3 Department of Microbiology, Calcutta School of Tropical Medicine, Kolkata, India; 4 Faculty of Tropical Medicine, Mahidol Oxford Research Unit, Mahidol University, Bangkok, Thailand; 5 Centre for Tropical Medicine, Churchill Hospital, Oxford, United Kingdom; 6 Faculty of Tropical Medicine, Department of Molecular Tropical Medicine and Genetics, Mahidol University, Bangkok, Thailand; 7 Center for Emerging and Neglected Infectious Diseases, Mahidol University, Bangkok, Thailand; Kenya Medical Research Institute - Wellcome Trust Research Programme, Kenya

## Abstract

**Background:**

The *Plasmodium vivax* that was once prevalent in temperate climatic zones typically had an interval between primary infection and first relapse of 7–10 months, whereas in tropical areas *P.vivax* infections relapse frequently at intervals of 3–6 weeks. Defining the epidemiology of these two phenotypes from temporal patterns of illness in endemic areas is difficult or impossible, particularly if they overlap.

**Methods:**

A prospective open label comparison of chloroquine (CQ) alone versus CQ plus unobserved primaquine for either 5 days or 14 days was conducted in patients presenting with acute vivax malaria in Kolkata. Patients were followed for 15 months and primary and recurrent infections were genotyped using three polymorphic antigen and up to 8 microsatellite markers.

**Results:**

151 patients were enrolled of whom 47 (31%) had subsequent recurrent infections. Recurrence proportions were similar in the three treatment groups. Parasite genotyping revealed discrete temporal patterns of recurrence allowing differentiation of probable relapse from newly acquired infections. This suggested that 32 of the 47 recurrences were probable relapses of which 22 (69%) were genetically homologous. The majority (81%) of probable relapses occurred within three months (16 homologous, 10 heterologous) and six genetically homologous relapses (19%) were of the long latency (8–10 month interval) phenotype.

**Conclusions:**

With long follow-up to assess temporal patterns of vivax malaria recurrence, genotyping of *P.vivax* can be used to assess relapse rates. A 14 day unobserved course of primaquine did not prevent relapse. Genotyping indicates that long latency *P.vivax* is prevalent in West Bengal, and that the first relapses after long latent periods are genetically homologous.

**Trial Registration:**

Controlled-Trials.com ISRCTN14027467

## Introduction

*Plasmodium vivax* malaria is notoriously difficult to eliminate, largely because of relapses which are derived from activation of liver hypnozoites. The only effective radical treatments for *P.vivax* infections are 8-aminoquinoline antimalarials, all of which produce oxidant haemolyis. These drugs are potentially dangerous in areas where glucose 6 phosphate dehydrogenase deficiency (G6PDd) is common (i.e. most tropical countries). As a result although the 8-aminoquinoline primaquine is widely recommended, it is often not prescribed. The proportion of vivax infections which relapse varies considerably across the tropical world. India has most of the world’s *Plasmodium vivax* malaria [1]. There have been few prospective studies of relapse patterns in India [2]–[5]. More recent investigations have indicated low relapse rates, which was one reason why India and several other countries adopted a five day primaquine regimen for radical treatment of vivax malaria over the past half-century [6], [7]. Relapse rates can be underestimated if follow-up terminates before relapses emerge. Long latency *P.vivax* was prevalent over Europe, and much of Asia. In the temperate areas relapses usually occurred 7–10 months after the initial febrile illness. Further north there was usually no primary illness, and the first symptoms of vivax malaria occurred 7–10 months after sporozoite inoculation [8]. Although generally thought to be confined to temperate regions such as the Koreas, long latency *P.vivax* may be much more widespread throughout the tropics. It is certainly also prevalent in Central America, North Africa, the horn of Africa, the middle East, Afghanistan, Central Asia, the Indian sub-continent and China. The first *P.vivax* to be sequenced fully was a long latency parasite from El Salvador (*Sal 1*) [8]. Clear evidence for long latency in Indian *P.vivax* was documented in the first half of the twentieth century [8], [9], and is supported by more recent studies [3]. In order to assess the effectiveness of short course primaquine regimens, and to determine whether long latency *P.vivax* was present around Kolkata, a prospective comparative trial supported by parasite genotyping was conducted with up to 450 days patient follow-up.

## Methods

### Clinical Procedures

Non-pregnant patients aged over 3 years presenting with symptomatic vivax malaria to the Calcutta School of Tropical Medicine were recruited into an open-label randomized comparison of three drug regimens provided they or their care givers gave fully informed written consent and were willing to be followed for at least one year. A full history and clinical examination were recorded on a standard proforma. Before treatment a haematocrit and the NADPH spot test for G6PD deficiency was performed and patients were withdrawn from the primaquine arms if this was positive. Blood for parasite genotyping was stored in EDTA tubes at −20°C until DNA extraction. Parasite counts were determined by counting infected red blood cells per 1000 red blood cells in thin smears under microscopy, or calculating the parasite count per 200 white blood cells in thick smears stained with Giemsa.

The following three treatment regimens were evaluated.

1. chloroquine (25 mg base/kg total) only
2. chloroquine (25 mg base/kg total) followed by primaquine 0.25 mg base/kg/day for five days
3. chloroquine (25 mg base/kg total) followed by primaquine 0.25 mg base/kg/day for 14 days

The first dose was observed but all subsequent antimalarial doses were to be taken at home. Each patient was instructed on the need to complete the full course of treatment.

Patients were seen daily until afebrile then weekly for one month and thereafter every one to two months for 15 months. If vivax malaria recurred blood was taken for parasite genotyping and comparison with the original infection, and patients were treated with chloroquine followed by primaquine 0.25 mg base/kg daily for 21 days.

The protocol for this trial and supporting CONSORT checklist are available as supporting information; see Checklist S1 and Protocol S1.

### Ethics Review

This study which was approved by the Ethics committee of the Faculty of Tropical Medicine, Mahidol University, and also by the Calcutta School of Tropical Medicine. The trial was registered with Controlled Clinical trials (CCT-NAPN-21654): ISRCTN14027467.

### Parasite Genotyping

*Plasmodium vivax* genomic DNA was extracted from 1 ml of venous blood using QIAampDNA kit (QIAGEN, Germany) according to the manufacturer’s instructions. Eight microsatellite (3.27, 11.162, 6.34, 8.504, 3.503, 14.297, 1.501, and 3.502) and three antigenic markers [10]–[13] were genotyped in 90 admission samples to assess genetic diversity. Three polymorphic microsatellite and three polymorphic antigen molecular markers were selected based on sensitivity (i.e. reliability of PCR amplification at the low parasite densities usually found in recurrent infections) in order to compare primary infections and recurrences (N = 39). Segments of genes encoding three antigenic markers, circumsporozoite protein (CSP), merozoite proteins 1 and 3 (MSP1, and MSP3), were assessed as described previously [10]–[12]. The PCR and RFLP bands were scored by comparison with reference samples (K2, K104, and Sal 1). Paired primary infection and corresponding recurrence samples were always run side by side. When multiple bands in PCR products were found, they were counted as multiple alleles. The probability that these arose from the same inoculation (genetically related) or different inoculations was then calculated based on background population frequencies as described below.

For microsatellite analysis, the lengths of the PCR products were measured in comparison to internal size standards (Genescan 500 LIZ) on an ABI 3100 Genetic analyzer (PE Applied Biosystems), using GENESCAN and GENOTYPER software (Applied Biosystems) to measure allele lengths and to quantify peak heights. Multiple alleles were called when there were multiple peaks per locus and where minor peaks were >33% of the height of the predominant allele. The presence of multiple alleles at any locus indicated a multiple genotype infection and the probability that these arose from the same or different mosquito inoculations was calculated as described below. The number of alleles, their distributions, and the degree of heterozygosity (He) were analysed to assess genetic diversity.

### Therapeutic Responses

Parasite clearance time (PCT) was calculated as the time taken for admission parasitaemia to fall below detectable levels. Fever clearance time (FCT) was the time until the patient’s temperature returned to <37.5°C following admission and remained below this for more than 48 hours.

### Statistical Methods

Variables were assessed by analysis of variance or the Chi square test as appropriate and relapse proportions were assessed by survival analysis using Stata®. Linear regression was used to assess the temporal pattern of recurrent infections. A best fit to the temporal pattern of late genetically heterologous recurrences was extrapolated back to study entry to provide an estimate of the new infection rate (conceding that some of these could have been relapses from an inoculation before that causing the incident infection in the current study). The excess of early heterologous recurrences were attributed to relapse.

All naturally acquired malaria infections comprise a family or families of parasites resulting from recombination of related or unrelated gametes. The probabilities that two genotypes arose from the same or different inoculations were calculated from the individual allele probabilities derived from the background study patient population (90 patients’ isolates from this study including all patients with recurrent infections and a random sample of the remainder who did not experience recurrence). Homologous genotype infections were infections in which all of the PCR amplified alleles were similar, or one or two of the microsatellite loci were different by a single repeat (suggesting PCR slippage) and all other loci were similar [13], and the probability that the two infections were acquired separately was statistically highly improbable (P<0.01).

## Results

**Figure 1:**
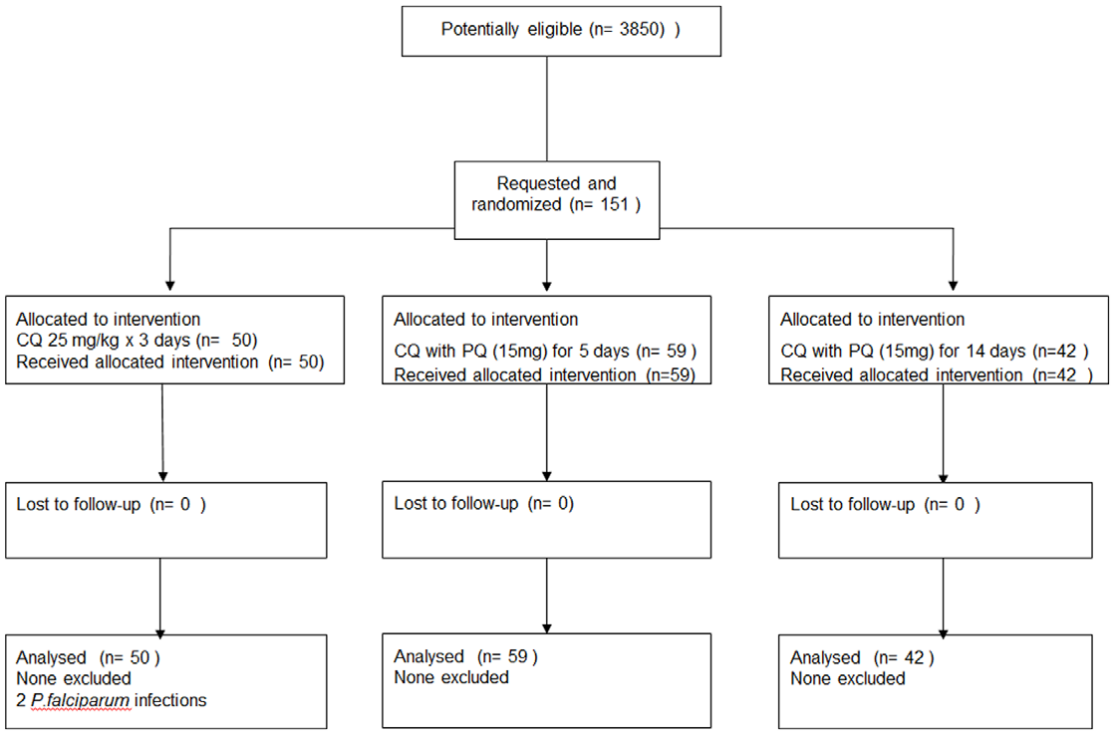
CONSORT flow chart.

A total of 151 patients with acute vivax malaria who were willing to comply with study procedures and the follow-up were enrolled between April 2003 and September 2004, and they completed follow- up at 1–2 monthly intervals for one year (Figure 1). All patients tolerated their medication well and recovered following antimalarial treatment. The treatments were generally well tolerated and were no serious adverse effects. The mean (range) fever clearance time was 2.5 (2–3) days and the mean parasite clearance time was 3.1 (3–4) days. There were no significant differences in admission characteristics or in FCT and PCT between the three treatment groups (Table 1).

**Table 1:** Patients’ admission characteristics.

Treatment regimen	Chloroquine alone	Chloroquine + 5 days primaquine	Chloroquine + 14 days primaquine
Number of patients enrolled	50	59	42
Male: female	47∶3	55∶4	37∶5
Mean (SD) age (years)	29 (10)	29 (11)	31 (12)
Median (range) No of previous malaria episodes	2 (0–6)	2 (0–11)	2 (0–9)
Mean (SD) duration of fever (days)	4 (4)	4 (4)	5 (9)
Temperature (°C)	37.5 (0.6)	37.7 (0.7)	37.6 (0.7)
Haematocrit (%)	38 (5)	38 (4)	39 (5)
G6PD deficiency	2	–	–
Hepatomegaly (%)	5 (10%)	3 (5%)	2 (5%)
Splenomegaly (%)	9 (18%)	5 (8%)	3 (7%)
Geometric mean (range) Parasite count (/uL)	4,320(80–25,680)	5,400(320–21,680)	3,280(40–19,040)
Mean (range) parasite clearance time (days)	3.0 (2–4)	3.2 (2–4)	3.1 (2–4)
Mean (range) fever clearance time (days)	2.5 (2–3)	2.6 (2–3)	2.5 (2–3)

### Recurrent Infections

**Figure 2:**
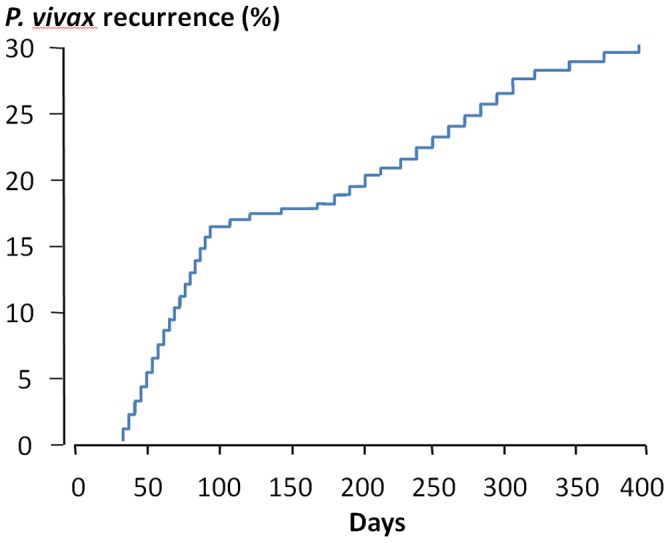
The temporal pattern of the 47 recurrences of *Plasmodium vivax* malaria in 151 patients with acute vivax malaria in Kolkata following treatment with chloroquine +/− primaquine. The three treatment groups were pooled as there were no significant differences in recurrence proportions between them.

There were 47 (31%) recurrent *Plasmodium vivax* infections among the 151 patients enrolled. None of these recurrences occurred within 28 days, the period recommended for the surveillance of chloroquine resistance (Figure 2). There was a clear biphasic pattern with approximately 60% of the recurrences occurring within three months. There were no differences in mean age, previous malaria episodes, fever duration before admission, physical examination findings, admission body temperature, haematocrit, and parasite count between the 47 patients with a recurrence of *P.vivax* malaria and those (N = 101) who had no recurrence. Two patients had multiple reappearances of vivax malaria and two patients presented with falciparum malaria 2 to 3 months after the reappearance of vivax malaria. There were no significant differences in the overall reappearance proportions or pattern of recurrences of vivax malaria between the three treatment groups; chloroquine (CQ) alone (N = 15; 31%), CQ plus 5 days primaquine (N = 16; 27%), CQ plus 14 days primaquine (N = 16; 38%); *P* = 0.61. Cure rates assessed at three months were also similar.

### Genetic Diversity

The number of alleles, and the heterozygosity of eight microsatellite and three antigenic markers in *P. vivax* population were high (He = 0.586 to 0.957), indicating substantial genetic diversity in the overall study population (Table 2). The three microsatellite loci with the highest diversity and the three antigenic markers (CSP, MSP1, MSP3) with high efficiency amplification were selected for comparison of paired samples. Levels of genetic diversity in primary and recurrence samples were both high (He  = 0.799 to 0.969). The estimated prevalence of the haplotype containing all the most common alleles was 2 in 1000, so with this genotyping approach the probability that two genetically homologous isolates would occur independently was therefore ≤0.002. There is therefore sufficient genetic diversity in these parasite populations to make it very unlikely that an identical genotype would infect the same individual twice in succession [10]–[13].

**Table 2:** Patterns of allelic diversity in the Kolkata *P. vivax* population.

	Overall population (n = 90)			Initialinfection (n = 39)			Corresponding recurrence (n = 39)		
Marker^1^	No of Alleles	He^2^	most commonalleles^3^(%)	No ofAlleles	He^2^	most commonalleles^3^(%)	No ofAlleles	He^2^	most commonalleles^3^(%)
PVCS	9	0.737	8 (40)	7	0.799	8 (36)	7	0.795	8 (33)
PVMSP1	26	0.918	4 (15)	22	0.955	1 (10), 18 (10)	23	0.969	18 (13)
PVMSP3	34	0.939	1 (17)	19	0.903	1 (26)	18	0.895	1 (26)
14.297	11	0.846	195 (22), 198 (22)	7	0.812	195 (28)	8	0.835	195 (26)
1.501	13	0.911	104 (18)	14	0.928	104 (18)	15	0.886	104 (26)
3.502	10	0.855	152 (21)	7	0.825	152 (31)	8	0.842	152 (28)
3.27	28	0.957	183 (11)						
11.162	5	0.628	184 (44)						
6.34	14	0.915	158 (18)						
8.504	11	0.782	205 (31)						
3.503	4	0.586	208 (58)						
**Mean (SD)**	14 (±10.01)	0.765(±0.25)		12.67(±6.71)	0.87(±0.07)		12.83(±6.11)	0.87(±0.06)	

1. Three antigenic and 8 microsatellite genetic loci.

2. He: heterozygosity.

3. The alleles were numbered and the number of the most commonly identified allele and the corresponding proportion (%) of all identified alleles are shown. When two are equal, both are shown.

Single genotypes were found in 54% of *P.vivax* infections in the study population overall and in 77% of recurrence isolates (P<0.001). Minor alleles or multiple genotype patterns were found randomly throughout the isolates, and most of the minor peaks were found as predominant peaks in other isolates. No particular associations of predominant and minor alleles were seen which might indicate PCR artifacts or linkage. Taken together these data suggested that the minor alleles observed represented genuine multiple genotype infections and not artefacts.

### Genotyping of Primary and Recurrent Infections

**Figure 3:**
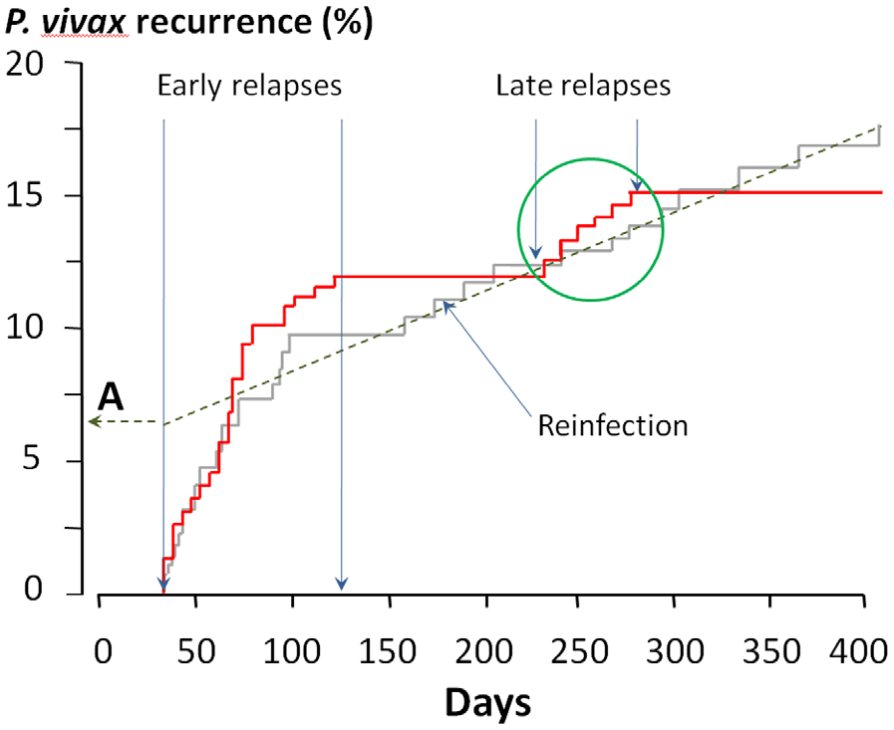
The same data as shown in **Figure 2** are now divided into genetically homologous relapses (shown in red) and genetically heterologous recurrences (shown in grey). The date of enrollment is shown as day 1 (recruitment was over a 5 month period). Heterologous recurrences occurring after 120 days were considered as reinfections allowing back-extrapolation of the proportion of all such recurrences which were suspected reinfections (point A) and thus by subtraction the proportion that were suspected relapses. The cluster of late genetically homologous relapses (green circle) presumably represent relapses of the long latency *P.vivax* phenotype.

Using the six genetic markers, no infections in different patients were found to be similar. Of the 47 initial infections which recurred subsequently 6 (13%) were with mixed genotypes. Compared with the initial infection 22 (46%) of the 47 recurrences were genetically homologous (there was only sufficient DNA to assess all six markers in 39 patients’ recurrences –in the other 8 three markers were assessed). The remaining 25 (54%) recurrences were genetically heterologous [11]–[14] (Table 3). Thus the cumulative proportions of genetically homologous and genetically heterologous recurrences were not significantly different *(*p = 0.20*)* (Figure 3). There were no clinical or laboratory differences between patients who subsequently had genetically homologous and genetically heterologous recurrent infections, and proportions of homologous relapse were similar in the three treatment groups; chloroquine (CQ) alone (N = 7/50; 14%), CQ plus 5 days primaquine (N = 9/59; 15%), CQ plus 14 days primaquine (N = 9/42; 21%) p>0.2. Thus there was no evidence that the unobserved primaquine regimen reduced the probability of subsequent recurrence.

**Table 3:** *P.vivax* recurrent infections in Kolkata.

Patients	Number
1. Patients with no recurrence	104
2. Patients with short latency homologous relapses	16
3. Patients with short latency suspected heterologous relapses	10
4. Patients with long latency homologous relapses	6
5. Patients with suspected new infections during 15 months follow up period	15
Total	**151**

Inspection of the temporal pattern of homologous recurrences revealed clear separation between early [mean (SD) interval from initial presentation: 69 (31) days, N = 16] and late relapses coinciding approximately with the start if the following year’s rainy season: [mean interval 255 [21] days; N = 6]. After treatment the genetically homologous and heterologous recurrences occurred at approximately similar rates for the first 100 days (Figure 3). Then there was a four month period during which there were no homologous recurrences followed by a tight cluster of six late homologous relapses (Data S1). The genetically heterologous recurrences occurred at a constant rate after the first 100 days, which was slower than the initial rate.

### Calculation of Relapse Proportions

As none of the recurrent *P.vivax* infections occurred within 28 days of presentation it is unlikely that chloroquine resistance resulting in recrudescence was a significant factor in determining the risk of recurrence [15], [16]. This suggests that recurrences were either relapses or reinfections. As reinfection with a similar genotype had a probability ≤0.002, recurrences of the same genotype were considered all to be relapses. The recurrences with different genotypes presumably resulted from both relapses and reinfections. It was assumed that genetically heterologous early relapses shared similar periodicity to the genetically homologous relapses in relation to the primary infection [11]–[13], whereas reinfections occurred at a much lower constant rate after four months and did not have any periodicity. The early pattern of both homologous and heterologous recurrences is very similar to the well described relapse pattern of “tropical” *Plasmodium vivax* [8]. A clear inflection in the temporal pattern of recurrences was evident four months after admission (Figure 2). After this point the rate of recurrence with different genotypes flattened and remained constant for almost one year, while there were no further genetically homologous relapses for four months. Given their lack of periodicity and constant rate the genetically heterologous recurrences after >4 months were therefore considered to be reinfections. Assuming a constant rate of reinfection reaching patency after decline in blood chloroquine concentrations below suppressive levels, a best fit linear relationship could then be back- extrapolated to the onset of the true relapses to provide an estimate of the total number of reinfections (point A, Figure 3). From this it can be deduced that of the 25 recurrences with heterologous genotypes, 10 (40%) were relapses and 15 (60%) were reinfections. With this estimate the revised true relapse numbers were 22+10 = 32 or 21% in the patients enrolled, of which 31% (N = 10) were genetically heterologous and 19% (N = 6) were of the long latency phenotype. Homologous and heterologous relapses occurred at approximately similar rates within three months of presentation. It should be noted that the initial infection in this study could also have been a relapse from earlier sporozoite inoculation, and that apparent new infections could be homologous long latency relapses from sporozoite inoculations preceding that which caused the incident infection in the study.

## Discussion

The overall *Plasmodium vivax* malaria recurrence rate in this study was 31%. At the time of this study the Calcutta School of Tropical Medicine treated around 6,000 malaria cases annually, of which approximately 65% were caused by *P. vivax*. The patients were mainly from urban, and a few were from peri-urban Kolkata. The absence of any recurrences within 28 days suggests vivax malaria in this area continued to be chloroquine-sensitive [16], [17], so recurrences were either caused by relapse or reinfection. In this open randomized comparison neither primaquine regimen reduced the incidence of *P.vivax* recurrence. The most likely explanation for this lack of effectiveness is poor adherence to the 14 day primaquine regimen, and lack of efficacy of the shorter course regimen [6], [7]. Although follow up was good in this study we cannot exclude asymptomatic self- limiting relapse, so it is possible that the true rate of relapse was higher than documented here.

Genotyping of recurrent vivax malaria indicates that in endemic areas relapse can be genetically homologous, genetically related (from a recent recombination event), or genetically unrelated [10]–[13]. It has been suggested that heterologous relapses (which occur at the same time as homologous relapses) result from activation of dormant hypnozoites by the acute infection [8]. In infants, who cannot have dormant liver hypnozoites, the first vivax recurrence of life is usually genetically homologous, which suggests that heterologous relapse resulting from a mixed genotype infection which was undetected in the primary infection is unusual [13]. Distinguishing a genetically unrelated relapse from a newly acquired infection in an endemic area with certainty is not possible, but can be inferred from temporal patterns [8]. Genotyping revealed two distinct patterns of homologous relapse in this study. The majority of recurrences occurred with a pattern similar to that observed throughout South-East Asia, with a mean interval from start of treatment of two months (16 genotypically homologous, 10 heterologous) but 6 of the 22 homologous relapses (27%) were clustered together and occurred after an interval of 7 to 10 months (Figure 3). This is exactly the interval expected of long latency *P.vivax.* Warrington Yorke studying the malariatherapy of neurosyphilis was first to note the importance of long follow up in characterizing relapse patterns. Following mosquito transmitted *P.vivax* of probable Indian origin in 1924 he considered initially that the relapses all occurred within two months of the initial infection [18], but continued observation revealed clustering of relapses 8–10 months after initial infection [9]. The extensively studied “Madagascar strain” had similar characteristics [19] as did the McCoy and St Elizabeth strains in the United States [8]. Adak et al have described both short and long latency vivax malaria coexisiting in Delhi -1300 kilometres to the NorthWest of Kolkata [3]. In the current series homologous and heterologous recurrences occurred at a similar rate for the first 3–4 months. The majority of these may have been activated by the initial infection [8]. Thereafter heterologous recurrences occurred at a much slower and constant rate suggesting these were all reinfections. There was no evidence of an increase in the rate of genetically heterologous recurrence 8–10 months later when the long-latency homologous relapses occurred. Extrapolation of the heterologous recurrences from the onset of relapses (reflecting decline of blood chloroquine levels below minimum inhibitory concentrations) allowed estimation of the proportion of heterologous recurrences which were relapses (10/25∶40%) and which were reinfections (15/25∶60%). Thus the estimated “true” relapse rate was 21% overall, of which 69% were genetically homologous, although this is a small series, and these estimates are therefore relatively imprecise. Parasite genotyping in vivax malaria has generally been regarded as uninformative, but this study illustrates that with sufficient follow-up, it can be used to estimate true relapse proportions, and that whereas short interval relapses maybe either genetically homologous or heterologous, the long latency relapses are all genetically homologous. Further studies in Asia, Central America and North Africa to investigate whether this pertains elsewhere are now needed.

This study provides strong evidence that long latency *P.vivax* is prevalent in West Bengal. The proportion of infections which have the long latency phenotype is almost certainly underestimated in this study as some may well also have had a short interval relapse, and all patients who had relapses were prescribed a 21 day course of primaquine (although adherence may well have been poor). It is also possible that many of the heterologous relapses were infections of the long latency phenotype, acquired over six months before presentation, which were activated by the primary illness [8]. Indeed the apparent primary infections in this series may well have comprised some relapses.

These observations have important implications for the epidemiological assessment of vivax malaria, and the evaluation of radical treatment, and the assessment of the likely impact of interventions. Further information from the Indian sub-continent is needed as this area harbours the majority of the world’s *P.vivax* burden. There has been no evidence of long latency vivax in tropical South East Asia but further studies there are needed. These observations also provide some support for the “activation of latent hypnozoites” (ALH) hypothesis [8]. In the ALH hypothesis the febrile illness randomly activates pre-existing and recently inoculated hypnozoites to cause initial relapses, which can be genetically homologous or heterologous, whereas the initial long latency relapse results from intrinsic “biological clock” activation, and as a consequence these late relapses are all genetically homologous [8].

## Supporting Information

Checklist S1 **Consort trial checklist.**
(PDF)

Protocol S1 **Trial protocol.**
(DOC)

Data S1 **Genotype details of long latency relapses.**
(DOCX)
