# Supplementary material for: Genotyping of Plasmodium vivax Reveals Both Short and Long Latency Relapse Patterns in Kolkata
Source: PLoS One. 2012 Jul 13;7(7):e39645. doi: 10.1371/journal.pone.0039645 (PMC3396609; doi:10.1371/journal.pone.0039645)
Supplement: Protocol S1 — Trial protocol. (DOC) [file pone.0039645.s002.doc]

**A randomised trial to assess the effectiveness of different primaquine regimens for the radical treatment of vivax malaria in Kolkota**

| Principal Investigator: | Dr JR Kim |
| --- | --- |
| Sponsor | Faculty of Tropical Medicine, Mahidol University |
| Funder | Wellcome Trust |

**Protocol development team:** Dr JR Kim, Prof A Nandy, Prof S Pukrittayakamee, Prof NJ White, Dr M Imwong

Confidentiality Statement

This document contains confidential information that must not be disclosed to anyone other than the Sponsor, the Investigator Team, host institution, relevant ethics committee and regulatory authorities.

List of abbreviations

CRF Case record form

DSMB Data and Safety Monitoring Board

G6PD Glucose-6-phosphate dehydrogenase

GCP Good Clinical Practice

Hct Haematocrit

PCR Polymerase Chain Reaction

PCT Parasite Clearance Time

SAE Serious Adverse Event

SYNOPSIS

| Study Title | A randomised trial to assess the effectiveness of different primaquine regimens for the radical treatment of vivax malaria in Kolkota |
| --- | --- |
| Trial Design | Open-label randomised trial to assess the relapse rates of *P. vivax* malaria in patients in Kolkota with two different primaquine regimens. |
| Trial Participants | Patients with acute uncomplicated *P. vivax* malaria |
| Sample size | 50 patients per drug arm. Estimated total sample size 150. |
| Inclusion Criteria | - Male or female, aged from 3 years to 65 years old, inclusive - Acute uncomplicated *P. vivax* malaria, confirmed by positive blood smear - Fever defined as > 37.5°C tympanic or oral temperature or a history of fever within the last 24 hours - Written informed consent (by legally acceptable representative in case of children) - Willingness and ability of the patients/guardians to comply with the study protocol for the duration of the study |
| Exclusion Criteria | - Signs of severe/complicated malaria (WHO, 2000) - Haematocrit < 25% or Hb < 8 g/dL at enrollment - Acute illness other than malaria requiring treatment - For females: pregnancy, breast feeding - Patients who have received chloroquine within the previous 7 days - History of allergy or known contraindication to trial drugs. - Severe G6PD deficiency |
| Planned Trial Period | 24 to 30 months (January2003 – June 2005) |
| Primary Objective | To compare the effectiveness of primaquine 0.25mg base/kg/day given for 5 days versus 14 days with a standard chloroquine regimen in *P*. vivax malaria. |
| Secondary Objectives | - To measure the clinical and parasitological responses with chloroquine alone and two chloroquine –primaquine regimens. - To assess safety and tolerability |
| Primary endpoint | Recurrence rates over 12-15 months follow-up. |
| Secondary endpoints | - Parasite clearance time assessed by microscopy - Fever clearance time (i.e. the time taken for the tympanic temperature to fall below 37˚C and remain there for at least 24 hours) - Genotype assessments |

BACKGROUND AND RATIONALE

*Plasmodium vivax* malaria is notoriously difficult to eliminate, largely because of relapses which are derived from activation of liver hypnozoites. The only effective radical treatments for *P.vivax* infections are 8-aminoquinoline antimalarials, all of which produce oxidant haemolyis. These drugs are potentially dangerous in areas where glucose 6 phosphate dehydrogenase deficiency (G6PD) is common (i.e. most tropical countries). Primaquine is widely recommended, but often not prescribed. The proportion of vivax infections which relapse varies considerably across the tropical world. India has most of the world’s *Plasmodium vivax* malaria (1). There have been few prospective studies of relapse patterns in India (2-5). More recent investigations indicated low relapse rates, which was one reason why India adopted a five day primaquine regimen for radical treatment of vivax malaria over the past half-century (6, 7). But relapse rates can be underestimated if follow-up terminates before relapses emerge. Long latency *P.vivax* was prevalent over Europe, and much of Asia. In the temperate areas relapses usually occurred 7 to 10 months after the initial febrile illness. Further north there was usually no primary illness, and symptoms occurred 7-10 months after sporozoite inoculation. Although generally thought to be confined to temperate regions such as the Koreas, long latency *P.vivax* is likely be much more widespread throughout the tropics. It is certainly also prevalent in Central America, North Africa, the horn of Africa, the middle East, Afghanistan, Central Asia, the Indian sub-continent (3) and China. Clear evidence for long latency in Indian *P.vivax* was documented in the first half of the twentieth century (8), and is supported by more recent studies (3). In order to assess the effectiveness of short course primaquine regimens, and to determine whether long latency *P.vivax* was present around Kolkata, a prospective comparative trial supported by parasite genotyping was conducted with up to 450 days follow-up.

**OBJECTIVES**

*Primary Objective*

To compare the effectiveness of primaquine 0.25mg base/kg/day given for 5 days versus 14 days together with a standard chloroquine regimen in *P*. vivax malaria.

*Secondary Objectives*

To measure the clinical and parasitological responses with chloroquine alone and two chloroquine –primaquine regimens.

To assess safety and tolerability of the different regimens

**TRIAL DESIGN**

Study sites

*Summary of Trial Design*

This will be a prospective open label comparison of chloroquine (CQ) alone versus CQ plus 5 days unobserved primaquine or CQ plus 14 days unobserved primaquine in patients with acute vivax malaria in Kolkota. Patients will be followed for 15 months and primary and recurrent infections were genotyped.

Study duration; **2 years**

*Primary and Secondary Endpoints*

Primary Endpoint

Recurrence rate of vivax malaria over 15 months

Secondary Endpoints

Parasite clearance time assessed by microscopy

Fever clearance time (i.e. the time taken for the tympanic temperature to fall below 37˚C and remain there for at least 24 hours)

Genotype assessments Parasite clearance time assessed by microscopy

*Trial Participants*

Overall Description of Trial Participants

Male and non-pregnant female patients aged between 36 months and 65 years with acute uncomplicated vivax malaria are the target study population. All study patients must meet the applicable inclusion and exclusion criteria.

I*nclusion Criteria*

- Male or female, aged from 36 months to 65 years old, inclusive
- Acute uncomplicated *P. vivax* malaria, confirmed by positive blood smear with asexual forms of *P. vivax*
- Fever defined as > 37.5°C tympanic temperature or a history of fever within the last 24 hours
- Written informed consent (by legally acceptable representative in case of children)
- Willingness and ability of the patients/guardians to comply with the study protocol for the duration of the study

*Exclusion Criteria*

- Signs of severe/complicated malaria (WHO, 2000)
- Haematocrit < 25% or haemoglobin (Hb) < 8 g/dL at enrollment
- Acute illness other than malaria requiring treatment
- For females: pregnancy, breast feeding
- Patients who have received antiamalrial treatment within the previous 7 days
- History of allergy or known contraindication to study drugs
- G6PD deficiency;patients who are deficient on spot testing will not be allocated to the two primaquine groups.

***Procedures***

*Informed Consent*

The subject (or witness if illiterate) or the parent or guardian of a minor must personally sign and date the latest approved version of the informed consent form before any study specific procedures are performed. Written and verbal versions of the participant information and informed consent in the local language will be presented to the participants detailing no less than: the exact nature of the study; the implications and constraints of the protocol; the known side effects and any risks involved in taking part. It will be clearly stated that participation is voluntary and that the participant or guardian is free to withdraw from the study at any time for any reason without prejudice to future care, and with no obligation to give the reason for withdrawal.

Written informed consent will be obtained by means of participant or guardian dated signature or thumb print (if unable to write) and dated signature of the person who presented and obtained the informed consent.

*Screening, Eligibility and Baseline Assessments*

Patients who present at the participating sites will be screened to assess eligibility. Full consent/assent will be obtained before any enrolment procedures are conducted. It will be made clear from the outset that refusal to participate will not jeopardize subsequent antimalarial treatment.

*Demographics and Medical History*

Basic demographic and epidemiological data (e.g. sex, age, address, prior treatment and a full medical history) will be recorded by the study staff.

*Physical Examination and Vital Signs*

Physical examination will be conducted by a qualified investigator. Weight, pulse, blood pressure, respiratory rate, temperature, spleen and liver size will be recorded.

*Drug history*

All prescribed or over-the-counter and traditional medications used within the last 7 days will be recorded. Any drug allergies will be recorded.

*Screening blood & urine tests*

- A parasite count from Giemsa stained thick and thin blood films
- Full blood count or haematocrit
- G6PD spot test

*Randomisation and blinding*

Subjects who fulfil all the inclusion criteria and have none of the exclusion criteria will be randomised 1:1;1 to one of the three treatment arms. Allocation will be done by drawing the next sequential numbered opaque envelope, which contains the study number of the patient referring to the study treatment. The subjects will be assigned a study arm through a computer-generated randomisation scheduleThis is an open-label study so the blinding of investigators and patients is not applicable. However, the randomisation procedure allows for adequate drug allocation concealment before envelopes are opened. All laboratory investigations will be performed without knowledge of the treatment allocation.

*Blood sampling*

On admission

On study admission, immediately before drug administration, blood will be collected for the following:

- parasite count
- EDTA 2mL whole blood sample for PCR parasite genotyping
- haematocrit

G6PD spot test

*Study drug regimens*

(1) chloroquine (25mg base /kg total) only

(2) chloroquine (25mg base /kg total) followed by primaquine 0.25mg base/kg/ day for five days

(3) chloroquine (25mg base/kg total) followed by primaquine 0.25mg base/kg/ day for 14 days

*For recurrences:*

chloroquine (25mg base /kg total) followed by primaquine 0.25mg base/kg daily for 21 days.

The first dose will be observed but all subsequent antimalarial doses will be taken at home. Each patient will be instructed on the need to complete the full course of treatment.

*Follow up procedures*

Patients will be seen daily until a febrile then weekly for one month and thereafter every one to two months for 15 months. If vivax malaria recurs blood will be taken for parasite genotyping and comparison with the original infection.

*Additional visits*

Patients presenting to the clinic with a fever or other symptoms on unscheduled days will be assessed by the study physician. Their temperature will be recorded and blood smear will be made for any patient with a documented fever (oral or tympanic temperature ≥ 37.5˚C) or a history of fever. Patients will be treated as clinically indicated.

Patients with recurrent parasitaemia

Patients with a recurrent parasitaemia during follow up will have blood taken for the following:

- parasite speciation and count
- haematocrit
- if parasitaemic: EDTA 2mL whole blood sample for PCR parasite genotyping

*Discontinuation/ Withdrawal of Participants from the Study*

Each participant has the right to withdraw from the study at any time. In addition, the investigator may withdraw a participant if he or she considers it necessary for any reason including:

- Significant non-compliance with study requirements
- An adverse event which requires discontinuation of the study medication or results in inability to continue to comply with study procedures
- Disease progression which requires discontinuation of the study medication or results in inability to continue to comply with study procedures
- Blood transfusion
- Development of severe malaria
- Loss to follow up

If an individual is prematurely discontinued from study treatment for any reason, the investigator will make every effort to perform the following evaluations: physical examination and vital signs assessment, haematocrit level, parasite count and AE assessment. The reason for withdrawal will be recorded in the CRF. If the subject is withdrawn due to an adverse event, the investigator will arrange for follow-up visits until the adverse event has resolved or stabilised.

**STUDY DRUGS**

Storage of Study Drugs

All efforts will be made to store the study drugs in accordance with the manufacturers' recommendations in a secure area.

Compliance with Study Drugs

Only the first dose will be administered as Directly-Observed-Therapy. If the patient vomits, and is re-dosed; this will be recorded in the CRF. This is an effectiveness not an efficacy study.

Concomitant Medication

Throughout the study, investigators may prescribe any concomitant medications or treatments deemed necessary (e.g. antipyretics or anti-emetics) to provide adequate supportive care except for antibiotics with antimalarial activity unless unavoidable (e.g. doxycycline, azithromycin). If these are required the patients will be kept in the study and this will be noted as a protocol deviation.

Antimalarials for recurrent infections (see Rescue treatment) and non-falciparum malaria (if applicable) will be prescribed as described above. Any medication, other than the study medication taken during the study will be recorded in the CRF.

**SAFETY REPORTING**

This trial will be using standard drugs that have either been registered for decades and evaluated extensively. Therefore, the safety aspects of this trial will be limited to close observation while patients are in hospital, treating any intercurrent illnesses or drug-related side effects and recording and reporting only serious adverse events.

Definitions

*Adverse Event (AE)*

In this study, an AE is either a drug-related side effect or a new or exacerbation of a pre-existing symptom, sign or illness (other than malaria).

Serious Adverse Event

*A serious adverse event is an AE that:*

results in death

is life-threatening i.e. the patient was at risk of death at the time of the AE

requires inpatient hospitalisation or prolongation of existing hospitalisation

results in persistent or significant disability/incapacity or

is a congenital anomaly/birth defect

requires acute medical or surgical care to prevent one of the outcomes listed above

Reporting Procedures for Serious Adverse Events

All SAEs will be reported by the site investigator to the Medical Monitor, Professor Nicholas White, or his designee, within one day of his or her awareness of the SAE..

The site investigator must also report the SAEs to the local ethics committee in accordance with local requirements.

**STATISTICAL considerations**

Sample size justification

The sample size was calculated based on an assumption that relapse rates would be 30% in the chloroquine only group and 4% in the 14 day primaquine group.

To allow for 20% loss to follow up or incomplete data, a total of 50 patients per arm will be recruited to detect such a difference with 95% confidence and 80% power. The groups will be compasred by survival analysis..

Statistical Analyses

Proportional data

These will be compared using chi squared or Fisher’s exact test, as appropriate. Crude proportions will be calculated with the exact 95% confidence intervals (CI), where relevant.

Continuous data

These will be summarised by medians (IQR, ranges) and means (standard deviations, 95% CIs), as appropriate, and will include the parasite counts and laboratory parameters. Comparisons of continuous data will be assessed using the paired/unpaired t tests or the sign rank/Mann Whitney U tests, as appropriate.

**ETHICS**

Declaration of Helsinki

The Investigator will ensure that this study is conducted in compliance with the Declaration of Helsinki

ICH Guidelines for Good Clinical Practice

The Investigator will ensure that this study is conducted according to any National Regulations and that it will follow the principles of the ICH Guidelines for Good Clinical Practice 1996.

Approvals

The study protocol and its associated documents will be submitted to the appropriate local ethics committees for written approval.

The Investigator will submit and, where necessary, obtain approval from the above parties for all substantial amendments to the original approved documents.

Risks

This study will use drugs that have been studied thoroughly and their toxicities are well described. In general, they are all well tolerated.

Benefits

Benefits of Treatment

Malaria is a disease that needs to be treated promptly. All patients will benefit from receiving efficacious treatment at no cost. They will be followed up closely and will be given rescue treatment if clinically indicated.

Alternatives to Study Participation

Subjects are able to decline freely participation in this study. If so, they will receive standard care for their malaria.

Confidentiality

The trial staff will ensure that the participants’ anonymity is maintained. All documents will be stored securely and be accessible to trial staff and authorised personnel only.

**DATA HANDLING AND RECORD KEEPING**

All study data will be recorded on a standard Case Report Forms (CRF. The participants will be identified by a study specific participant number and/or code in any database. The name and any other identifying detail will NOT be included in any study data electronic file. Data may be used alone or in combination with data from related studies in secondary analyses.

**SPONSORSHIP**

The Faculty of Tropical Medicine is the study sponsor.

**PUBLICATION POLICY**

Any data published in the peer-reviewed medical literature will protect the identity of the subjects. This trial will be registered in a web based protocol registration scheme. All those who have made a substantial contribution will be co authors on publications.

**REFERENCES**

1. Mendis K, Sina BJ, Marchesini P, Carter R. **The neglected burden of *Plasmodium vivax* malaria.** *Am J Trop Med Hyg* 2001; 64(1-2 Suppl): 97-106.
2. Srivastava HC, Sharma SK, Bhatt RM, Sharma VP. **Studies on *Plasmodium vivax* relapse pattern in Kheda district, Gujarat.** *Indian J Malariol* 1996; **33:** 173–179
3. Adak T, Sharma VP, Orlov VS. **Studies on the *Plasmodium vivax* relapse pattern in Delhi, India.** *Am J Trop Med Hyg* 1998; **59:** 175-9.
4. Adak T, Valecha N, Sharma VP. ***Plasmodium vivax* polymorphism in a clinical drug trial.** *Clin Diagn Lab Immunol* 2001; **8:** 891-4.
5. Gogtay NJ, Desai S, Kadam VS, Kamtekar KD, Dalvi SS, Kshirsagar NA. **Relapse pattern of *Plasmodium vivax* in Mumbai: a study of 283 cases of vivax malaria.** *J Assoc Phys India* 2000; **48:** 1085-6.
6. Gogtay NJ, Desai S, Kamtekar KD, Kadam VS, Dalvi SS, Kshirsagar NA. **Efficacies of 5- and 14-day primaquine regimens in the prevention of relapses in *Plasmodium vivax* infections.** *Ann Trop Med Parasitol* 1999; **93:** 809-12.
7. Sinha S, Dua VK, Sharma VP. **Efficacy of 5 day radical treatment of primaquine in Plasmodium vivax cases at the BHEL industrial complex, Hardwar.** *Indian J Malariol* 1989; **26:** 83-6.
8. Yorke W . **Further observations on malaria made during the treatment of general paralysis.**  *Trans R Soc Trop Med Hyg 1925 108-130.*
