# Supplementary material for: Genotyping of Plasmodium vivax Reveals Both Short and Long Latency Relapse Patterns in Kolkata
Source: PLoS One. 2012 Jul 13;7(7):e39645. doi: 10.1371/journal.pone.0039645 (PMC3396609; doi:10.1371/journal.pone.0039645)
Supplement: Data S1 — Genotype details of long latency relapses. (DOCX) [file pone.0039645.s003.docx]

Data S1: Supporting information

Typing of the three microsatellite and three antigen gene loci in initial admission and the late relapses.

| Patient code | ms14.297  (3 nucleotides)) | |  | ms1.501 (7nucleotides) | |  | ms3.502  (8nucleotides) | CSP | MSP1 | MSP3 | Relatedness |
| --- | --- | --- | --- | --- | --- | --- | --- | --- | --- | --- | --- |
| K47 | 195 |  | | 118 |  | | 152 | b vk210 no/no | a a2 m2 | a al1 h1 |  |
| K47R | 195 |  | | 118 |  | | 152 | b vk210 no/no | a a2 m2 | a al1 h1 | Identical |
| K92 | 198 |  | | 111 |  | | 168 | a vk210 no/no | b a1 m1 | a al1 h1 |  |
| K92R | 198 |  | | 111 |  | | 168 | a vk210 no/no | b a1 m1 | a al1 h1 | Identical |
| K93 | 198 | 201 | | 104 |  | | 144 | b vk210 no/in | b a5 m1 | a al1 h1 |  |
| K93R | 195 |  | | 104 |  | | 144 | b vk210 no/in | a a5 m1 | a al1 h1 | Related |
| K108 | 201 |  | | 104 |  | | 152 | b vk210 in/in | b a1 m2 | b al1 h2 |  |
| K108R | 201 |  | | 104 |  | | 152 | b vk210 in/in | b a1 m2 | b al1 h2 | Identical |
| K112 | 195 |  | | 97 |  | | 152 | b vk210 no/in | b a2 m2 | a al1 h4 |  |
| K112R | 195 |  | | 97 | 181 | | 152 | b vk210 no/in | b a2 m2 | a al1 h4 | Identical |
| K127 | 198 |  | | 104 |  | | 152 | a vk210 no/in | a a7 m4 | a al4 h3 |  |
| K127R | 198 |  | | 104 |  | | 152 | a vk210 no/in | a a7 m4 | a al4 h3 | Identical |
